# Alabama

**Published:** 1896-10

**Authors:** 


*Alabama. Bulletin No.* 72 of the Alabama Agricultural Experiment Station has recently been issued relating to the various skin-tumors of horses and mules. It is illustrated, and refers to some experimental work.

